# Antimicrobial Food Packaging Based on Prodigiosin-Incorporated Double-Layered Bacterial Cellulose and Chitosan Composites

**DOI:** 10.3390/polym14020315

**Published:** 2022-01-13

**Authors:** Lúcia F. A. Amorim, Cláudia Mouro, Martijn Riool, Isabel C. Gouveia

**Affiliations:** 1 FibEnTech Research Unit, Faculty of Engineering, University of Beira Interior, 6200-001 Covilhã, Portugal; lucia.amorim@ubi.pt (L.F.A.A.); d1684@ubi.pt (C.M.); 2 Department of Medical Microbiology and Infection Prevention, Amsterdam UMC, Amsterdam Institute for Infection and Immunity, University of Amsterdam, 1105 AZ Amsterdam, The Netherlands; m.riool@amsterdamumc.nl

**Keywords:** bacterial cellulose, prodigiosin, antimicrobial, bacterial pigments, electrospinning, food packaging

## Abstract

Nowadays, food packaging systems have shifted from a passive to an active role in which the incorporation of antimicrobial compounds into biopolymers can promote a sustainable way to reduce food spoilage and its environmental impact. Accordingly, composite materials based on oxidized-bacterial cellulose (BC) and poly(vinyl alcohol)-chitosan (PVA-CH) nanofibers were produced by needleless electrospinning and functionalized with the bacterial pigment prodigiosin (PG). Two strategies were explored, in the first approach PG was incorporated in the electrospun PVA-CH layer, and TEMPO-oxidized BC was the substrate for nanofibers deposition (BC/PVA-CH_PG composite). In the second approach, TEMPO-oxidized BC was functionalized with PG, and afterward, the PVA-CH layer was electrospun (BC_PG/PVA-CH composite). The double-layer composites obtained were characterized and the nanofibrous layers displayed smooth fibers with average diameters of 139.63 ± 65.52 nm and 140.17 ± 57.04 nm, with and without pigment incorporation, respectively. FTIR-ATR analysis confirmed BC oxidation and revealed increased intensity at specific wavelengths, after pigment incorporation. Moreover, the moderate hydrophilic behavior, as well as the high porosity exhibited by each layer, remained mostly unaffected after PG incorporation. The composites’ mechanical performance and the water vapor transmission rate (WVTR) evaluation indicated the suitability of the materials for certain food packaging solutions, especially for fresh products. Additionally, the red color provided by the bacterial pigment PG on the external surface of a food packaging material is also a desirable effect, to attract the consumers’ attention, creating a multifunctional material. Furthermore, the antimicrobial activity was evaluated and, PVA-CH_PG, and BC_PG layers exhibited the highest antimicrobial activity against *Staphylococcus aureus* and *Pseudomonas aeruginosa*. Thus, the fabricated composites can be considered for application in active food packaging, owing to PG antimicrobial potential, to prevent foodborne pathogens (with PG incorporated into the inner layer of the food packaging material, BC/PVA-CH_PG composite), but also to prevent external contamination, by tackling the exterior of food packaging materials (with PG added to the outer layer, BC_PG/PVA-CH composite).

## 1. Introduction

Production and abusive use of packaging materials from fossil fuels is leading to increased environmental concerns, especially the short-term application of single-use plastic, such as in food packaging [1]. Most of this single-use synthetic plastic packaging is disposed of in the environment and not recycled, with incineration or landfilling frequently used as end-of-life treatments, and a large part of the plastics end up in water bodies, where they are broken down into microplastics by ultraviolet radiation and physical forces [1,2,3]. Moreover, fragmented synthetic plastics contain toxic substances, such as plasticizers, pose a hazardous risk to the ecosystem and human health [3]. Hence, to mitigate the synthetic plastic waste-related issues, the growing interest in biopolymers and their application for the development of biodegradable packaging solutions has intensified [4,5]. Concerning the biopolymers used as the basis for obtaining biodegradable materials, several options have been evaluated regarding both, synthetic biopolymers, such as polyhydroxyalkanoates [6,7], polylactic acids [8], and poly-(butylene succinate) [9], or natural biopolymers, such as chitosan (CH) [10,11], gelatin [12,13], alginate [14], starch [15], or bacterial cellulose (BC) [16,17]. BC is already produced as a nanomaterial, with remarkable intrinsic properties, namely the high porosity combined with the high surface area and biodegradability [18]. Thus, BC and its derivatives have been extensively explored in the development of biodegradable packaging, namely as a reinforcing agent [19], or to produce edible films [20], and even to the production of active and intelligent food packaging materials, with the addition of bioactive compounds [17,21], as well as additives in electrospun nanofibers for different purposes [22]. Additionally, the reactive groups within its structure provide numerous possibilities for modifications [23]. The modification of the BC surface allows the modulation and enhancement of the multifunctional biopolymer characteristics without altering its bulk properties [24,25]. Oxidation is one of the most widely employed chemical modifications on the BC surface. The oxidation can be carried out by nitroxyl-based oxidizers, the most commonly used is 2,2,6,6-tetramethyl-1-piperidinyloxy (TEMPO), which directly functionalizes the primary alcohols with carboxyl groups, increasing the negative surface charge density [23]. 

Chitosan (CH), a cationic polysaccharide, mainly obtained from crustacean shells, is the second most abundant biopolymer, after cellulose, and has incredible applications owing to its biocompatibility and biodegradability [11,26,27]. Additionally, its viscosity, solubility, ion binding, gas and aroma barrier properties, and antimicrobial ability, as well as its easy modification due to its reactive functional groups, make CH a versatile polymer for a wide range of applications, including the food packaging field [26,28,29]. Nevertheless, most common physical forms of CH (such as flakes, powder, and films) exhibit low surface area, restricting its applicability. Therefore, CH-based nanomaterials, presenting a variety of morphologies, have been gaining increased attention [30,31]. Nanofibers emerged as an appealing morphology, especially due to the high specific surface area and high porosity. Electrospinning is a commonly used fiber spinning technique where a high voltage is applied to create an electrically charged jet of a polymer solution, producing the polymer fibers. Moreover, needleless electrospinning technology, in which polymeric jets are created from liquid surfaces, using a rotating electrode, enables easy maintenance, stability, and higher production capacity, including production scale-up from laboratory settings to an industrial level [32,33,34]. 

Besides the extensive research regarding biopolymers for the development of novel packaging systems, there is also increased attention towards the addition of antimicrobial agents, to inhibit food spoilage or pathogenic microorganisms and to extend the shelf-life of packaged goods [35,36,37,38]. Antimicrobial peptides, essential oils, and organic acids are natural antimicrobial compounds often applied in active food packaging systems [39,40,41]. Furthermore, other bioactive agents, such as natural pigments, extracted from different sources, also exhibit compatible features to be explored as antimicrobial agents, including biodegradability, non-toxicity, and non-carcinogenicity [42,43,44,45]. For instance, the bacterial pigment prodigiosin (PG), a secondary metabolite widely produced by several species, including *Serratia* sp., displays advantageous bioactive properties including anticancer, antimalarial, antifungal, immunosuppressive, and antimicrobial activity against several pathogenic bacteria, including foodborne pathogens, as previously reported by Arivizhivendhan and collaborators, using meat extract powder as a model food material [46,47]. Furthermore, the higher efficacy of PG antibacterial properties has also been reported with the synergic combination of prodigiosin with antibiotics and biosurfactants [47]. Moreover, unlike synthetic dyes, natural pigments are biodegradable, non-toxic, non-carcinogenic, and several efforts have been made towards their cost-effective production through the identification of new bacterial sources, utilization of low-cost substrates, and optimization of process parameters [48].

As aforementioned, the main purpose of providing antimicrobial properties to food packaging materials is to inhibit foodborne pathogens and avoid food spoilage. Another important aspect, not so often explored, is the protection of the external surface of food packaged goods. The exterior surface could be a potential vehicle for the transmission of bacteria and an increased possibility of cross-contamination during food processing [49,50,51]. Thus, an opportunity to further enhance food packaging as a barrier against external contaminants should not be overlooked. 

**Figure 1:**
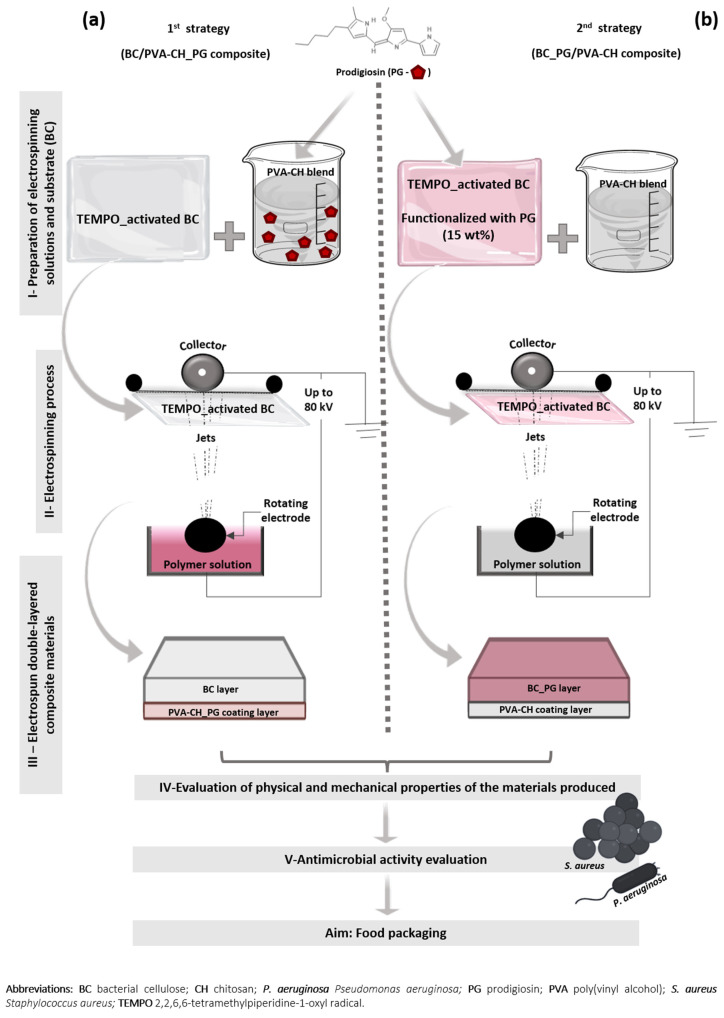
Schematic representation of the production strategies used in composites preparation (**a**) BC/PVA-CH_PG composite, with TEMPO-activated BC, used as a substrate for PVA-CH_PG nanofibers deposition and (**b**) BC_PG/PVA-CH composite, with PVA-CH nanofibers deposition in TEMPO-activated BC, previously functionalized with PG.

Therefore, this work aimed to produce two different double-layered materials, with different characteristics, which would be suitable for different food packaging applications. To the best of our knowledge, PG has never been tested as an antimicrobial compound in exterior or interior surfaces of food packaging applications. Accordingly, both materials were produced using BC, PVA, CH, and the bacterial pigment PG, applying two different production strategies. One of the strategies was to use TEMPO-activated BC as a substrate for PVA-CH_PG nanofibers deposition, producing a BC/PVA-CH_PG composite material. On the other strategy, TEMPO-activated BC was functionalized with PG and used as a substrate for the coating layer, made of PVA-CH nanofibers, producing a double-layer BC_PG/PVA-CH material (Figure 1).

## 2. Materials and Methods

### 2.1. Materials

Kombucha Original Bio, a commercial beverage that contains live bacteria and yeast species able to produce bacterial cellulose (BC) [52], was obtained from Freshness Diagonal, Lda (Freshness Diagonal, Lda, Montijo, Portugal). Poly(vinyl alcohol) PVA (115,000 g/mol) was purchased from VWR Chemicals (VWR Chemicals, Leuven, Belgium). Glacial acetic acid, sodium chloride (NaCl), ethanol, and dipotassium hydrogen phosphate were provided by Fisher Scientific (Fisher Scientific, Leicestershire, UK). Chitosan (CH) (low molecular weight), sodium hydroxide, peptone, Nutrient Agar (NA), Brain Heart Infusion Agar (BHI), hydrochloric acid (HCl), glycerol, glucose monohydrate, 2,2,6,6-tetramethylpiperidine-1-oxyl radical (TEMPO), sodium bromide (NaBr), and sodium hypochlorite (NaOCl) were purchased from Sigma-Aldrich (Sigma-Aldrich, St. Louis, MO, USA). Agar-agar was acquired from Labkem (Labkem, Barcelona, Spain).

### 2.2. BC Production and Recovery

BC production was accomplished using 10% (*v*/*v*) pre-inoculum of Kombucha Original Bio commercial beverage. The fermentation bioreactor, besides the pre-inoculum, contained 8.25 g/L of commercial green tea, 8.25 g/L of commercial black tea, and 70 g/L glucose. The fermentation was carried out for seven days, under static conditions, at 30 °C. After production, the pellicles were recovered and submitted to a washing procedure (0.1 M NaOH, at 80 °C, for 30 min), for impurities and cellular debris removal [53,54]. Thereupon the pellicles were rinsed with distilled water to decrease the pH from the alkali washing procedure and dried at room temperature until a constant weight was achieved. 

#### 2.2.1. Preparation of Oxidized BC

The oxidation system TEMPO/NaBr/NaClO was used to oxidize hydroxyl groups of BC to introduce a negative surface charge. Briefly, 4 g of dry BC were placed in 100 mL of a previously prepared solution containing 0.0125% (*w*/*v*) TEMPO, 0.125% (*w*/*v*) NaBr, and 3.2% (*v*/*v*) NaClO, in alkaline medium (pH 10.5, adjusted with 1 M NaOH) and stirred for 60 min. Then, 0.1 M HCl was used to decrease the pH of the solution to 7, and the pre-activated BC was washed in deionized water. 

### 2.3. Production and Recovery of PG Pigment

*Serratia plymuthica* was gently provided by Peter Askew (Industrial Microbiological Services Ltd, Hampshire, UK). The bacterium was selected for PG pigment production, which was accomplished in Peptone Glycerol Phosphate (PGP) medium (5 g/L peptone, 10 mL/L glycerol, 2 g/L K_2_HPO_4_, and 15 g/L agar, for solid growth) [45]. The fermentation was carried out at 20 °C, with the absence of light. The pigment extraction was performed accordingly with a previously reported method [46] using acidified ethanol. 

### 2.4. Fabrication of the Double-Layered Composite Materials

Both composite materials were produced using BC, CH, PVA (blended with CH to facilitate its spinnability [55,56,57]) and the bacterial pigment PG, applying two different production strategies (Figure 1):

1st strategy (BC/PVA-CH_PG composite): the previously TEMPO-activated BC was used as a substrate for PVA-CH_PG nanofibers deposition. To produce this nanofibrous coating layer, a modified electrospinning method, Nanospider technology (Nanospider laboratory machine NS LAB 500S, Elmarco, Liberec, Czech Republic) was used. The layer was produced with 10% (*w*/*v*) PVA and 2% (*w*/*v*) CH, with the polymer solutions prepared separately, and blended in a ratio of 70:30, with also 15 wt% of PG incorporated in the blend (by PVA-CH blend weight). The PVA solution was prepared with distilled water and the CH solution in 0.1 M glacial acetic acid, with pH adjusted to 5 using 0.1 M HCl, producing a positive electrostatic charge. This blend was electrospun, on top of the pre-activated BC layer, by applying a voltage of 75 kV, an electrode spin of 45 Hz, and a working distance of 15 cm, at room temperature.

2nd strategy (BC_PG/PVA-CH composite): PVA-CH nanofibers deposition in TEMPO-activated BC, functionalized with 15% PG at 40 °C, during 60 min, 20 rpm, with a raise velocity of 2 °C/min, and liquid ratio 1:30 in an AHIBA IR dyeing machine (AHIBA IR, Datacolor company, Lawrenceville, NJ, USA). The PVA-CH blend was prepared and electrospun using the same parameters as in the 1st strategy but without PG pigment incorporation. 

### 2.5. Characterization of the Double Layer Composite Materials

#### 2.5.1. Scanning Electron Microscopy (SEM)

The morphology of the electrospun nanofibers and the double-layered composites was examined using a scanning electron microscope (S-3400N, Hitachi, Tokyo, Japan) after having been sputtered with gold using Quorum Q150R ES sputter coater (Q150R ES, Quorum Technologies Ltd, Laughton, UK), for conductivity. All SEM experiments were carried out at 20 kV. ImageJ software (Image J, National Institutes of Health, Bethesda, MD, USA) was used to calculate average fiber diameter and diameter distribution, from the SEM micrographs, by randomly selecting 100 nanofibers.

#### 2.5.2. Attenuated Total Reflectance–Fourier Transform Infrared Spectroscopy (ATR-FTIR) Analysis 

FTIR was performed for each layer produced, mainly to evaluate BC oxidation by TEMPO and PG incorporation. The infrared spectra were obtained in the region between 4500 and 600 cm^−1^, with 4 cm^−1^ resolution, using the attenuated reflectance modulus (ATR) in the Thermo-Nicolet is10 FT-IR spectrophotometer (Thermo-Nicolet is10 FT-IR spectrophotometer, Waltham, MA, USA). All readings were performed at room temperature and the infrared spectrum of each experimental sample was gathered by averaging 32 scans.

#### 2.5.3. Porosity Measurement

The porosity of each layer produced in this work was measured by the liquid displacement method. The dry weight of each sample was recorded, W_S_, as well as the weight of measuring cylinders filled with ethanol (W_1_). The samples immersed in ethanol, in the measuring cylinders, were then sonicated in a water bath at 30 °C, for 40 min to aid solvent penetration into the pores of the samples. Following this, the cylinders refilled with ethanol were weighed again (W_2_) and, once more after samples removal (W_3_). The porosity (ε) of the samples was calculated by the following equations:

(1) $$V_{s}=\left(W_{1}-W_{2}+W_{s}\right)/\rho_{e}$$

(2) $$V_{p}=\left(W_{2}-W_{3}-W_{s}\right)/\rho_{e}$$

(3) $$\varepsilon \;\left(\%\right)=V_{p}/\left(V_{p}+V_{s}\right)\times 100$$

$$\Leftrightarrow \varepsilon \;\left(\%\right)=\left(W_{2}-W_{3}-W_{s}\right)/\left(W_{1}-W_{3}\right)\times 100$$

where ρ_e_ represents the density of ethanol, V_s_ is the volume of the sample, and V_p_ is the volume of the sample pores, as described elsewhere [58].

#### 2.5.4. Water Contact Angle (WCA) Measurements

The static WCAs of the layers produced were measured, at room temperature, using a Dataphysics Contact Angle System OCAH-200 (OCAH-200, DataPhysics Instruments, Filderstadt, Germany) apparatus to examine the surface wettability. Samples were placed on top of glass microscope slides and deionized water was used as reference fluid. Droplets of 4 µL were placed at different locations on the sample’s surface. Each reported contact angle was the mean value of at least 10 measurements. 

#### 2.5.5. Water Vapor Transmission Rate (WVTR)

The WVTR, through the double-layered BC/PVA-CH_PG and BC_PG/PVA-CH composites, was measured accordingly to the ASTM E96/E96M-15 standard method. Briefly, each composite sample was cut into a circular shape and used to seal the top of the glass test tubes (1.2 cm in diameter), containing 10 mL of deionized water. The sealing film was used around the opening of each test tube, where the samples were attached, to seal the sides and prevent any moisture loss. The composite materials were incubated at 37 °C, and periodic weighing was performed. Weight changes indicated the loss of water through the test samples and the WVTR was calculated according to the following Equation (4):

(4) $$\mathrm{WVTR}=\frac{W_{\mathrm{loss}}}{A}$$

where W_loss_ is the weight loss of water (daily), and A is the area of the test tube opening (m^2^).

#### 2.5.6. Color Measurement 

The color strengths (K/S values) of the layers functionalized with the PG pigment, i.e., BC_PG and PVA-CH_PG, were obtained using a Datacolor 110 spectrophotometer (Datacolor 110 spectrophotometer, Datacolor company, Lawrenceville, NJ, USA), under illuminant D65 using 10° standard observer. The K/S was calculated based on the Kubelka–Munk equation:

(5) KS=(1−R)22R

where R is the observed reflectance of the colored sample under the wavelength of 540 nm, K is the absorption coefficient and S is the scattering coefficient. The results were the average of at least five measurements at different positions.

#### 2.5.7. Mechanical Testing

To evaluate the mechanical performance of individual layers and the composite materials produced, tensile tests were performed on a Universal tensile test machine (DY-35, Adamel Lhomargy, Roissy en Brie, France), equipped with a 100 N static load cell. Samples were cut into strip-shaped specimens of 5 mm width and 30 mm long and stored in desiccators for at least 24 h prior testing. Tensile strength, Young’s modulus, and elongation at break were evaluated at 1 mm/min, as previously reported [59]. All measurements were performed for at least five specimens of each sample, and the average value was recorded.

### 2.6. Antibacterial Properties Evaluation

The ability of the PVA-CH, PVA-CH_PG, BC, and BC_PG layers to inhibit the bacterial growth of *Staphylococcus aureus* ATTC 6538 (*S. aureus*) and *Pseudomonas aeruginosa* PA25 (*P. aeruginosa*) was tested through the ASTM E2180-07 agar slurry test for antimicrobial surfaces. Bacterial suspensions of *S. aureus* and *P. aeruginosa*, grown in NA and BHI media, respectively, were prepared at ߤ10^8^ colony forming units (CFU)/mL and 1 mL of cells were added to 100 mL of sterile agar slurries (0.85% (*w*/*v*) NaCl and 0.3% (*w*/*v*) agar-agar). The inoculated agar slurries were then pipetted over the test samples and over filter paper with a 0.22 µm pore size, which acted as the control. To evaluate the antibacterial activity, the inoculated agar slurries were recovered from the samples immediately after inoculum application (0 h), and after 24 h in contact with the agar slurries, at 37 °C. The recovery of the inoculated agar slurries was accomplished by adding sterile saline solution (NaCl) to the inoculated samples and shaken vigorously to facilitate the release of the agar slurry. After recovery, at 0 and 24 h, serial dilutions were carried out, also with NaCl solution, placed on NA plates and incubated at 37 °C for 24 h. The percentage of bacterial reduction (%R) was calculated accordingly with Equation (6):

(6) Percentage Reduction (%R)=((C−S)/C)×100

where S represents the number of CFUs obtained with the samples and C is the CFUs of bacteria recovered from filter paper controls.

### 2.7. Statistical Analysis 

Each experience was performed at least three times unless otherwise stated. GraphPad Prism 6 software (GraphPad Prism 6, La Jolla, CA, USA) was used to perform a one-way analysis of variance (ANOVA), followed by Tukey’s multiple comparisons test. A *p*-value below 0.05 was considered statistically significant. All data were expressed as mean ± standard deviation (SD). 

## 3. Results and Discussion

### 3.1. Nanofibers Diameters and Morphology

**Figure 2:**
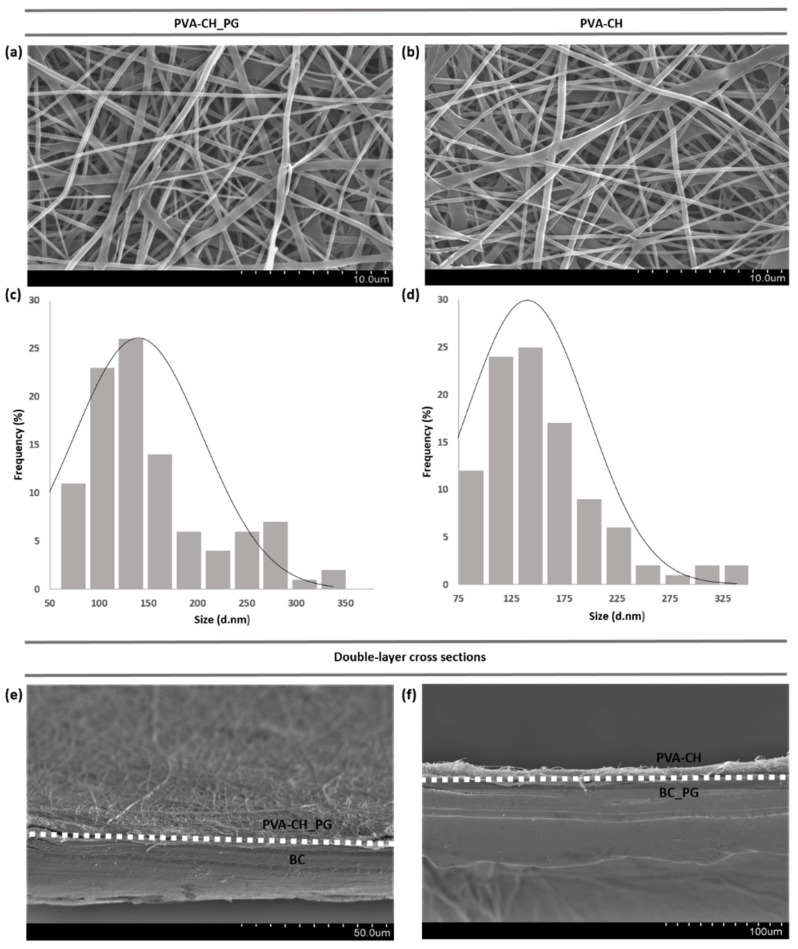
SEM images of PVA-CH_PG coating layer (**a**) and PVA-CH without PG pigment (**b**); fiber diameter distributions of PVA-CH_PG (**c**) and PVA-CH (**d**); cross-section of the composite materials produced, with a perspective emphasizing the layer containing the PG pigment: BC/PVA-CH_PG (**e**) and BC_PG/PVA-CH (**f**).

In the electrospinning process, fiber formation and morphology can be affected by several parameters, including solution properties (viscosity, polymer concentration, electrical conductivity, surface tension), processing conditions (applied voltage, distance from electrode to collector, volume feed rate, electrode type), and ambient conditions (temperature, humidity) [60,61]. Parameter optimization was performed in the needleless electrospinning system used in this work, to obtain electrospun materials with desirable final properties, including smooth morphology and low fiber diameters for increased surface area to volume ratio. SEM results revealed that the nanofibers arrangement of PVA-CH_PG (Figure 2b) was similar to that of PVA-CH (Figure 2a), mostly quite uniform with smooth surface and cylindrical shape, albeit some flat ribbon-like fibers morphologies could also be observed. Additionally, diameter distribution analysis (Figure 2c,d) demonstrated that the diameter of PVA-CH nanofibers (140.17 ± 57.04 nm) was nearly unchanged by PG pigment incorporation (PVA-CH_PG; 139.63 ± 65.52 nm). Moreover, Figure 2e,f show the cross-section of the composite materials produced, BC/PVA-CH_PG and BC_PG/PVA-CH, exhibiting the double-layered structure and displaying the surface of the layers containing PG pigment. 

### 3.2. FTIR Spectral Analysis

**Figure 3:**
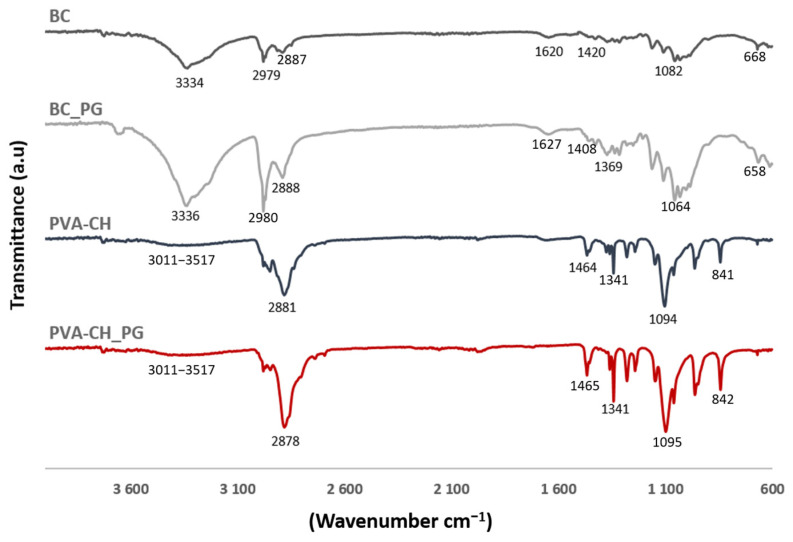
FTIR spectra of each composite layer, with (BC_PG and PVA-CH_PG) and without pigment incorporation (BC and PVA-CH).

FTIR_ATR was performed in each composite layer, with and without the PG pigment incorporated. The spectra obtained are shown in Figure 3, and BC spectra present a broad absorption peak centered at 3334 cm^−1^, which corresponds to O–H stretching vibration, the peak at 2979 cm^−1^ was attributed to C–H stretching vibration, CH_2_ asymmetric stretching was identified at 2887 cm^−1^, characteristic absorption bands appear at 1082 cm^−1^, assigned to C–C bonds of polysaccharide monomer units, the peak at 1420 cm^−1^ was attributed to symmetric CH_2_ bending vibration, and the infrared band observed at 668 cm^−1^ was assigned to C–OH out of plane bending [59,62,63]. The characteristic band of carboxylate (COO^–^) was identified at 1620 cm^−1^, indicating BC oxidation by TEMPO [23,64]. In PVA_CH spectra a broad, but low-intensity peak, is observed around 3011–3517 cm^−1^, assigned to O–H and N–H stretching vibrations, the peak at 2881 cm^−1^ was attributed to C–H stretching vibration, the band observed at 1464 cm^−1^ corresponds to amide groups of CH, also the band at 1341 cm^−1^ is attributed to C–O bond of CH, the absorption band at 1240 cm^−1^ is assigned to O–H bending vibration, C–O stretching vibration was identified at 1094 cm^−1^, and C–C stretching of PVA occurs at 841 cm^−1^ [65,66,67,68]. The spectra of both layers with PG, as shown in Figure 3, are similar to the aforementioned BC and PVA_CH spectra, with no distinct peak shifts, but presented an increased intensity at specific wavenumbers, corresponding to PG functional groups. The BC_PG and the PVA_CH_PG spectrum showed the characteristic peaks for PG at 1369 cm^−1^ and 1341 cm^−1^, respectively, corresponding to a pyrrole group, 2888 cm^−1^ and 2878 cm^−1^ corresponding to a methylene group, and 3336 cm^−1^ and 3011–3517 cm^−1^, assigned to the amide group [69]. Moreover, the lack of peaks shifts in the spectra of both layers containing PG, indicates the absence of changes in the chemical structure of PVA-CH_PG and BC_PG layers with the pigment incorporation, suggesting that PG is only adsorbed within the materials, without new chemical bonds formation.

### 3.3. Porosity Evaluation

The major advantages of nanofibers are their large surface area and high porosity, resulting in a wider application potential, including the possibility of incorporating compounds within the nanofibrous structure, which are protected from the external environment [70,71]. Therefore, the porosity of both layers, with and without incorporation of the PG pigment, was evaluated by the liquid displacement method. Ethanol was used as displacement liquid, once it easily penetrates into the pores without leading to shrinkage or swelling of the fibers [72]. The BC layer exhibited high porosity values (85.33 ± 5.23 %), Table 1, due to its web-like network structure [18,24,73], and was only slightly reduced after PG pigment incorporation (75.96 ± 2.86%). The porosity of the PVA-CH electrospun layer was also high (90.99 ± 3.85%) and remained unaffected after incorporation with the PG pigment (91.62 ± 5.16%). The layers’ high porosity and high surface area are desirable features for physical interaction with active compounds, such as antimicrobials [24,74]. Additionally, for specific applications, such as the PG controlled release, it is also possible to tailor BC’s porosity by varying fermentation conditions and post-treatment methods, as well as the nanofibers’ structure, during the electrospinning process [74,75]. 

### 3.4. Water Contact Angle Analysis

Composite layers hydrophobicity was evaluated through WCA measurements, and the results are displayed in Table 1. From the measured WCAs, the BC (37.40 ± 7.69°) and BC_PG layer (48.90 ± 7.33°) displayed moderate hydrophilic behavior. However, BC pellicles are usually characterized by higher hydrophilicity, due to the presence of intramolecular hydrogen bonds [76]. BC’s intrinsic properties could have been affected by the NaOH alkaline treatment, used to wash BC pellicles after fermentation [54], which leads to fiber swelling and surface area increase, crystallinity index decrease, and an intermolecular rearrangement of individual chains, usually followed by a decrease in hydrophilicity [77]. Another factor contributing to the higher WCA reported herein, for BC and BC_PG layers, may be the loss of hydrogen bonds while the carboxylate groups are formed, as reported in Section 3.2, during BC TEMPO-oxidation [78]. PVA-CH and PVA-CH_PG layers also exhibited moderate hydrophilic behavior with WCAs of 39.50 ± 11.04° and 35.50 ± 9.97°, respectively. Even though the hydrophobic behavior of CH films has been reported in other works, with WCAs > 88° [79,80], the blending with PVA, with very hydrophilic nature and WCAs < 10° [81], affects the wettability of the nanolayer produced. Furthermore, the decrease in the WCA with increasing PVA content in a PVA_CH blend has been previously described [79,82].

### 3.5. WVTR Measurements

Barrier properties, such as WVTR, play a major role in the development of materials intended for food packaging applications since barrier performance is a crucial factor determining the applicability range of such materials [83]. Therefore, the WVTR of both double-layered composite materials produced was evaluated. The results revealed a similar and high WVTR value, with 1113.71 ± 335.88 g/m^2^/day for BC/PVA-CH_PG and 888.98 ± 125.12 g/m^2^/day for BC_PG/PVA-CH. These results are comparable to those reported by other authors, 1389.25 g/m^2^/day for BC [84], and 1113.75 g/m^2^/day for a PVA-CH composite film [66] and could be attributed to the materials hydrophilicity, which leads to absorption of much more water vapor. The high WVTR values obtained will restrict the applicability of the materials produced for certain food packaging applications, such as respiring fresh products to control moisture evaporation [83,85]. Further improvements in the barrier properties of BC/PVA-CH_PG and BC_PG/PVA-CH will widen the composites’ applicability within the food packaging field.

### 3.6. Color Evaluation 

The red color provided by PG on the external surface of packaging material is also a desirable effect, to attract the consumers’ attention. Nonetheless, color evaluation was performed for each layer, before (BC and PVA-CH) and after functionalization with the PG pigment (BC_PG and PVA_CH_PG), and the results are summarized in Table 2. BC_PG exhibited the highest color strength (K/S value) at the maximum absorption wavelength, a thirteen-fold increase in K/S compared with the BC layer without pigment incorporation, whereas the PVA-CH_PG coating layer exhibited a smaller increase, of seven-fold, when compared with PVA-CH.

The different pigment incorporation procedures may play a significant role in contributing to the K/S differences observed, particularly the temperature parameter during the procedures. High K/S values were already reported for PG pigment, in dyed cellulosic fibers, with the increase in dyeing temperature [86] and BC functionalization was performed at 40 °C, whereas in the PVA-CH layer the PG incorporation into the polymers solution and the electrospinning process were performed at room temperature. 

Additionally, the TEMPO activation procedure modifies the BC fibrils, the introduction of carboxylate groups promotes the rupture of inter-chain hydrogen bonds which may cause higher accessibility to the inner fiber surface, enhancing pigment penetration [78,87].

Another important aspect affecting the color yield is the higher surface area of the electrospinning-produced coating layer compared with BC. The same effect was observed by Khatri and collaborators when comparing cellulose nanofibers with conventional cotton fiber [88].

### 3.7. Mechanical Properties

The mechanical performance evaluation was carried out for individual layers (BC and PVA-CH), as well as for the composite materials produced (BC/PVA-CH_PG and BC_PG/PVA-CH) and the results are summarized in Table 3. Regarding the BC layer, satisfactory mechanical properties were obtained with a tensile strength of 26.25 ± 2.79 MPa, and Young’s modulus of 186.63 ± 12.89 MPa, as expected, due to BC’s web-like structure, crystallinity index, and a high degree of polymerization [18,24,73,89].

On the other hand, the nanofibrous coating layer exhibited a much lower elastic modulus (22.41 ± 2.00 MPa), but higher elongation at break (84.70 ± 9.77%) once PVA_CH nanofibers were considerably less stiff than BC sheets, similar to preceding reports [90]. 

Nonetheless, the mechanical behavior observed for the composite materials, BC/PVA-CH_PG and BC_PG/PVA-CH, regarding the tensile parameters evaluated, was indicative of the BC layer major contribution for the composite’s improved mechanical performance, as expected, since cellulose and its derivatives have been extensively applied to obtain reinforced polymeric materials [22] The strengthened effect resulted in values of 26.11 ± 2.42 MPa and 26.50 ± 1.83 MPa, regarding the tensile strength, Young’s modulus of 172.01 ± 10.33 MPa and 182.71 ± 12.22 MPa, and an elongation at break of 15.17 ± 0.80% and 14.50 ± 0.10%, for BC/PVA-CH_PG and BC_PG/PVA-CH composites, respectively. The similarity between these results indicated that PG incorporation did not affect the materials’ mechanical performance. Moreover, according to conventional standards, the produced composite materials comply with the tensile strength requirements to potential food packaging applications [91,92].

### 3.8. Antibacterial Efficacy Evaluation

The bacterial inhibitory efficacy of BC, PVA-CH electrospun layer, and PVA-CH and BC layers with PG pigment incorporated was evaluated against *S. aureus* and *P. aeruginosa*, which are among the most commonly encountered foodborne bacteria [93].

**Figure 4:**
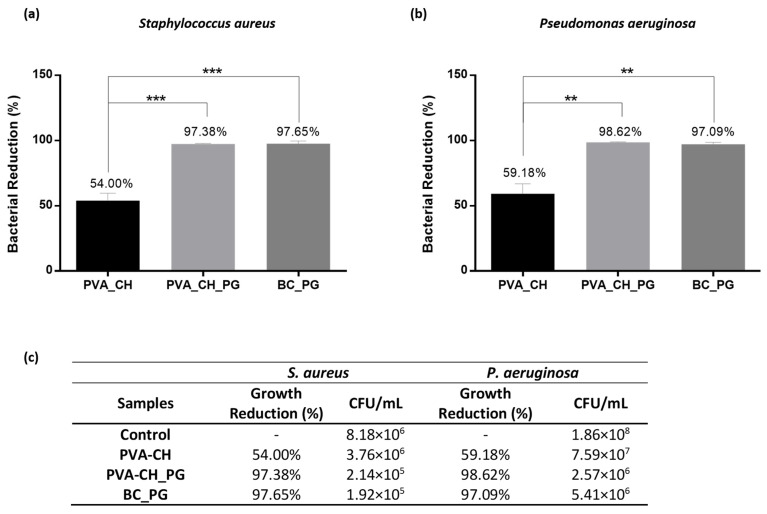
Evaluation of the antibacterial activity of PVA-CH coating layer, and both nanocomposite layers with PG pigment incorporated (PVA-CH_PG and BC_PG) against *S. aureus* (**a**) and *P. aeruginosa* (**b**). Correspondence between the percentage of bacterial reduction and the respective CFU/mL (**c**). (Values reported as mean ± SD, ** p < 0.01 and *** p < 0.001).

As expected, for the BC layer, no bacterial growth inhibition was observed, due to BC’s lack of inherent antibacterial activity [94]. In the case of the PVA-CH electrospun layer, some growth inhibition was observed for both bacteria investigated (Figure 4a,b). The bacterial growth inhibition is attributed to CH polysaccharide, whose antimicrobial activity was previously reported and several action mechanisms have been proposed [11]. One of the mechanisms more frequently suggested is the interaction of CH protonated amino groups with negatively charged membranes of bacteria, decreasing their permeability, which leads to cell leakage and death [57,95]. Moreover, the incorporation of prodigiosin pigment in the PVA-CH coating layer (i.e., PVA-CH_PG) revealed a significant pathogenic bacterial reduction, with 97.38 ± 0.57% growth inhibition for *S. aureus* and 98.62 ± 0.37% for *P. aeruginosa* (Figure 4a,b). This increased bacterial reduction is attributed to PG pigment’s ability to pass through the outer membrane and capability to disrupt the plasma membrane via a chaotropic-mediated mode faction [96,97]. The effect of PG incorporation is also evident in the BC layer, with growth inhibition of 97.65 ± 2.04% and 97.09 ± 1.54% for *S. aureus* and *P. aeruginosa*, respectively (Figure 4a,b). Moreover, the minimum inhibitory concentration (MIC) of PG pigment against the aforementioned pathogens was previously determined in our workgroup. The MIC value against *S. aureus* was found to be 0.24 mg/mL, while for *P. aeruginosa* the MIC value was 1.25 mg/mL [98]. Therefore, the increased bacterial reduction attributed to the PG presence in the fabricated materials was expected, since the concentration of PG used for the double-layered materials development was far superior to the MIC values obtained for each pathogenic bacteria studied.

Thus, the incorporation of PG in each composite layer increases the suitability of the fabricated materials for food packaging applications, with the ability to avoid food spoilage, and the possibility to prevent foodborne pathogens-related illnesses. Moreover, taking into account the antimicrobial activity obtained for each layer, different combinations could be anticipated to fabricate other materials specifically tailored. For example, the incorporation of PG pigment simultaneously in both layers, to evaluate any possible synergic effect, and layer-by-layer strategies, for prolonged antimicrobial effect.

## 4. Challenges and Future Research Directions

The development of novel systems such as the materials fabricated in this work, intended for food packaging applications, comes with several challenges that need to be addressed. At the economical level, the electrospinning technology used in this work, the needleless electrospinning method, will contribute to higher economic feasibility, without requiring further optimizations for the large-scale production of the materials proposed [34]. Nonetheless, regarding the microbial bioresources employed, some economic and technological challenges must be surpassed for the double-layer materials’ cost-effective production. Over the last years, efforts have been made to reduce production costs, by replacing synthetic media constituents with alternative feedstock, which would be a suitable strategy to obtain cost-effective bioresources, such as BC and PG pigment [48,99]. Moreover, the path for the cost-effective production of bacterial pigments may be the identification of new bacterial sources, such as actinobacteria, able to produce bioactive pigments that might diffuse into the medium, which ultimately facilitates pigment recovery and reduces the overall cost of the downstream process [100].

Regarding the composite materials herein reported, the performance of the materials accordingly with the intended food packaging conditions should be evaluated. Namely, evaluate PG pigment release and the duration of the antibacterial properties, evaluate the synergic effect of PG incorporation in both layers of the materials, assess the stability of the materials under standard food storage conditions (i.e., light exposure, and lower temperatures), and evaluate if the composite materials, besides the tensile strength required, also exhibit adequate mechanical stability to be applied in food packaging, by dynamic mechanical tests.

## 5. Conclusions

In this study, new BC/PVA-CH_PG and BC_PG/PVA-CH composites were successfully fabricated by needleless electrospinning technology. The double-layer materials were produced with coating layers presenting smooth fibers and low fiber diameters, 139.63 ± 65.52 for PVA-CH_PG nm and 140.17 ± 57.04 nm for PVA-CH nanofibrous layer, as needed for increased surface area for greater contact with food products, in case of application in active food packaging systems. The presence of carboxylate groups and, therefore successfully BC oxidation as confirmed by FTIR, and the CH positive electrostatic charge allowed the assembly of the coating layer directly on the BC surface. Increased intensity at specific wavelengths was also observed in the FTIR analysis, after PG incorporation, with no considerable peak shifts, indicating the absence of new chemical bonds formation. Moreover, the high porosity values obtained for each layer and the moderate hydrophilic behavior remained mostly unaffected after PG incorporation. However, the materials’ moderate hydrophilicity contributes to the high WVTR values obtained, which hinder the applicability of the materials produced for specific food packaging applications, where moisture evaporation is required. Moreover, the composites mechanical performance was majorly ascribed to the presence of BC, confirming its necessity to provide mechanical support to the coating layer. 

Thus, the composite materials produced display advantageous properties for potential applications in the food packaging field, with the BC_PG/PVA-CH composite showing promising results not only to address the packaging interior, due to its CH antibacterial properties but also with a multifunctional external surface, with antibacterial activity and color provided by PG, since a packaging material with a colored external surface is more appealing to consumers and the color evaluation of each composite layer revealed that BC_PG exhibited the highest color strength.

## Figures and Tables

**Table 1:** Porosity and water contact angle (WCA) values, obtained for each layer produced, with (PVA-CH_PG and BC_PG) and without (PVA-CH and BC) prodigiosin incorporation (data shown as mean ± SD).

	Porosity (%)	WCA (°)
**BC**	85.33 ± 5.23	37.40 ± 7.69
**BC_PG**	75.96 ± 2.86	48.90 ± 7.33
**PVA_CH_PG**	91.62 ± 5.16	35.50 ± 9.97
**PVA_CH**	90.99 ± 3.85	39.50 ± 11.04

**Table 2:** Apparent color, reflectance, and color strength (K/S value) of each layer before and after functionalization with PG.

	Apparent Color	Reflectance (%R)	K/S
**BC**	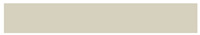	66.63	0.02
**BC_PG**	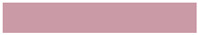	25.92	1.06
**PVA-CH**	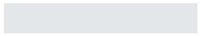	82.01	0.08
**PVA-CH_PG**	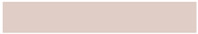	58.87	0.14

**Table 3:** Mechanical properties of the individual layers and the double-layered composites produced (data shown as mean ± SD).

	Young’s Modulus (MPa)	Tensile Strength (MPa)	Elongation at Break (%)
**BC**	186.63 ± 12.89	26.25 ± 2.79	14.15 ± 2.47
**PVA-CH**	22.41 ± 2.00	18.91 ± 1.83	84.70 ± 9.77
**BC/PVA-CH_PG**	172.01 ± 10.33	26.11 ± 2.42	15.17 ± 0.80
**BC_PG/PVA-CH**	182.71 ± 12.22	26.50 ± 1.83	14.50 ± 0.10

## Data Availability

Data that support the findings of this study are included in the article.

## Abbreviations

**BC** bacterial cellulose; **BHI** brain heart infusion; **CFU** colony forming units; **CH** chitosan; **HCl** hydrochloric acid; **MIC** minimum inhibitory concentration; **NA** nutrient agar; **NaBr** sodium bromide; **NaCl** sodium chloride; **NaOCl** sodium hypochlorite; **K/S** color strength; ***P. aeruginosa*** *Pseudomonas aeruginosa;* **PG** prodigiosin; **PGP** peptone glycerol phosphate; **PVA** poly(vinyl alcohol); ***S. aureus*** *Staphylococcus aureus;* **SEM** scanning electron microscopy; **TEMPO** 2,2,6,6-tetramethylpiperidine-1-oxyl radical; **WCA** water contact angle; **WVTR** water vapor transmission rate.
